# Functional and cooperative stabilization of a two-metal (Ca, Zn) center in α-amylase derived from *Flavobacteriaceae* species

**DOI:** 10.1038/s41598-017-18085-4

**Published:** 2017-12-20

**Authors:** Huijia Yin, Zhou Yang, Xinyu Nie, Shannan Li, Xuyang Sun, Chao Gao, Zenghang Wang, Guangming Zhou, Ping Xu, Chunyu Yang

**Affiliations:** State Key Laboratory of Microbial Biotechnology, Shandong University, Jinan, 250100 People’s Republic of China

## Abstract

Mesophilic α-amylase from *Flavobacteriaceae* (FSA) is evolutionary closely related to thermophilic archaeal *Pyrococcus furiosus* α-amylase (PWA), but lacks the high thermostability, despite the conservation of most residues involved in the two-metal (Ca, Zn) binding center of PWA. In this study, a disulfide bond was introduced near the two-metal binding center of FSA (designated mutant EH-CC) and this modification resulted in a slight improvement in thermostability. As expected, E204G mutations in FSA and EH-CC led to the recovery of Ca^2+^-binding site. Interestingly, both Ca^2+^- and Zn^2+^-dependent thermostability were significantly enhanced; 153.1% or 50.8% activities was retained after a 30-min incubation period at 50 °C, in the presence of Ca^2+^ or Zn^2+^. The C214S mutation, which affects Zn^2+^-binding, also remarkably enhanced Zn^2+^- and Ca^2+^- dependent thermostability, indicating that Ca^2+^- and Zn^2+^-binding sites function cooperatively to maintain protein stability. Furthermore, an isothermal titration calorimetry (ITC) analysis revealed a novel Zn^2+^-binding site in mutant EH-CC-E204G. This metal ion cooperation provides a possible method for the generation of α-amylases with desired thermal properties by *in silico* rational design and systems engineering, to generate a Zn^2+^-binding site adjacent to the conserved Ca^2+^-binding site.

## Introduction

In the field of biotechnology, there is an increasing interest in engineering proteins with enhanced thermostability and altered activity^1–3^. This requires a comprehensive understanding of the determinants of thermostability. By comparing the microscopic stability features of mesophilic and thermophilic protein homologs, various mechanisms underlying stabilization and adaptive mutations in thermophilic enzymes that determine the overall rigidity have been identified^4^, revealing common factors contributing to thermostability, such as the number of disulfide bonds, a high core hydrophobicity^5^, salt bridge formation^1^, metal-binding activity, ionic interactions^6,7^, and an improved quality of packing^8^.

Starch-hydrolyzing α-amylases (EC 3.2.1.1) are widely used for various industrial applications, e.g., in starch saccharification, textiles, food products, fermentation, the preparation of ethanol as fuel, as well as in clinical, medical, and analytical chemistry^9^. Based on the sequence similarity and classification in the Carbohydrate-Active Enzyme (CAZy) database (www.cazy.org)^10^, the vast majority of α-amylases belong to the glycoside hydrolase family 13 (GH13), which includes 40 subfamilies^11^. In agreement with their origins, most GH13_5 α-amylases are bacterial, but the GH13_6 and GH13_7 subfamilies include plant and archaeal α-amylases^12^. However, due to their close relationship with some thermophilic archaeal α-amylases^13^, some *Flavobacteriaceae* α-amylases have been assigned to GH13_7, e.g., the α-amylase FSA we described previously^14^.

With a general structure characterized by three domains, all GH13 α-amylases adopt a (β/α)_8_-barrel fold as a catalytic domain (i.e., domain A), with a catalytic triad formed by two consensus Asp residues and a Glu residue^15^. Based on a sequence alignment and site mutations, a short, conserved stretch covering the β_1_ strand of the catalytic (α/β)_8_-barrel has been found in many α-amylases, with the FYW compositions in archaeal α-amylases, FNW in plant-derived α-amylases, and FEW in animal-derived α-amylases^16^. Furthermore, Tyr39 in *Thermococcus hydrothermalis* has been demonstrated to contribute to thermostability^17^.

Thermostable (thermophilic) α-amylases are highly attractive for commercial use and have received increasing attention in industrial fields, and for studies of the physical mechanisms underlying the thermal stability of proteins^18^. The successfully resolved crystal structures of some *Bacillus* α-amylases, such as *Bacillus subtilis* (BSUA), *B. amyloliquefaciens* (BAA), and *B. licheniformis* (BLA)^19–21^, have led to the confirmation of various proposals regarding the stabilizing role of structural features^18^. A conserved Ca^2+^-binding site, located at the interface between domains A and B, confers protein stability. It is well-known that domain B is the least conserved region in the α-amylase family, with the most variation in length and sequence^22^. However, with a few exceptions, this Ca^2+^-binding site is conserved in most GH_13 family α-amylases, and is essential for retaining protein structure and catalytic activity^18,23^. Some Ca^2+^-independent α-amylases also have been reported, and these show great advantages compared to Ca^2+^-dependent α-amylases^24–26^. Crystallographic analysis of thermophilic and Ca^2+^-independent *P. furiosus* α-amylase (PWA) also uncovered a conserved Ca^2+^-binding site located at the interface between domains A and B^27^. Another metal ion, Zn^2+^, has also been detected near this Ca^2+^-binding site in domain B, and forms a novel two-metal center, i.e., a (Ca, Zn)-binding site. Mutagenesis of the cysteine (Cys166) in the Zn^2+^-binding site results in a drastic reduction of PWA catalytic activity at high temperatures^28^. Therefore, it is speculated that both sites of this two-metal center are involved in stabilizing the catalytically active conformation of PWA at high temperatures^27^. Based on a sequence alignment, a similar (Ca, Zn) center was also predicted in some hyperthermophilic α-amylases from *Thermococcus* species, such as the recently reported α-amylase from *Thermococcus* sp. HJ21^29^. However, the functional mode of this two-metal center, and potential synergistic effects are unclear.

In our previous work, a novel α-amylase, was identified from a novel *Flavobacteriaceae* species. Using the CAZy database^10^, FSA was assigned to the GH13_7 subfamily; it is phylogenetically related to archaeal PWA (sequence identity, 48%), but not to other bacterial α-amylases^14,30^. Based on a sequence alignment, most residues involved in Ca^2+^ and Zn^2+^ binding are conserved in FSA. In the present study, the stabilizing function of this two-metal site was investigated by constructing a series of mutants and by an isothermal titration calorimetry (ITC) analysis.

## Results

### Influence of Ca^2+^ and Zn^2+^ on the activity and thermostability of wild-type FSA

**Figure 1:**
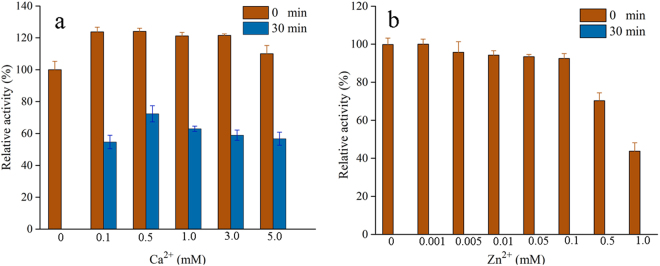
Influence of Ca^2+^ (**a**) or Zn^2+^ (**b**) on the FSA activity. Enzyme activity was measured after pre-incubation the starch-free reaction mixtures with 0.5 mM Ca^2+^, 0.01 mM Zn^2+^, at 50 °C for 0 min and 30 min, respectively. For relative activity calculation, the activity of FSA in the absence of metal ions and without pre-incubation was set as 100%.

The α-amylase activity of FSA was measured in the absence or presence of various concentrations of Ca^2+^ and Zn^2+^. As shown in Fig. 1a, the α-amylase activity of FSA was moderately enhanced by the addition of Ca^2+^; approximately 120% activity was detected in the presence of Ca^2+^ (ranging from 0.1 mM to 5 mM). To evaluate the thermostability, all KH_2_PO_4_-Na_2_HPO_4_ reaction mixtures were substrate-free and were incubated at 50 °C for 30 min, with the indicated concentrations of Ca^2+^ or Zn^2+^. In the absence of Ca^2+^, FSA showed the complete loss of activity after 30 min of incubation. Conversely, remarkably higher residual activities were detected in the presence of Ca^2+^ at concentrations of 0.1 to 5 mM. Supplementation with 0.5 mM Ca^2+^ resulted in the highest activity (74.5%) after 30 min of incubation. In contrast to the positive effect of Ca^2+^ on FSA activity and thermostability, only marginally increased activity was observed in the presence of low concentrations of Zn^2+^, and activity was remarkably inhibited in the presence of 0.5 mM Zn^2+^ (Fig. 1b).

### Influence of Ca^2+^ and Zn^2+^ on the thermostability of FSA mutants

**Figure 2:**
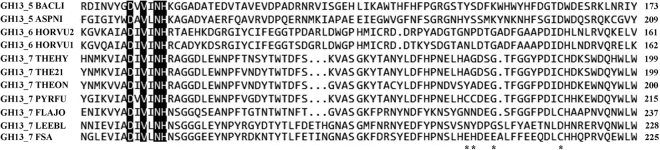
Multiple sequence alignment of FSA and amylases from GH13_5, 13_6, and 13_7 subfamilies by ClustalX. The mutated residues in this study are marked as *. *B. licheniformis*, BACLI; *Aspergillus niger*, ASPNI; *Hordeum vulgare*, HORVU1; *Hordeum vulgare*, HORVU2; *T. hydrothermalis*, THEHY; *T*. sp. HJ21, THE21; *T. onnurineus*, THEON; *P. furiosus*, PYRFU; *Flavobacterium johnsoniae*, FLAJO; *Leeuwenhoekiella blandensis*, LEEBL; FSA, present study.

With the aim of reconstructing the two-metal (Ca, Zn) center observed in PWA and enhancing thermostability, a series of mutations involving Ca^2+^ and Zn^2+^ binding sites were evaluated (Fig. 2). Obviously, these mutations did not influence protein expression; clear, single bands of 52 kDa were detected by sodium dodecyl sulfate (SDS)-polyacrylamide gel electrophoresis (PAGE) (Figure S1), indicating that the expression levels for all constructs were similar to those FSA.

It has been reported that a disulfide bond adjacent to the Zn^2+^-binding site was engaged between Cys163 and Cys164 in PWA^27^. This disulfide bond was unusual in the sequences included in the comparative analysis, as shown in Fig. 2, and was suspected to be functional in maintaining the rigidity of PWA at higher temperatures^27^. In this study, we first introduced a disulfide bond by constructing a mutant of EH-CC, in which Glu200 and His201 in FSA were both replaced with Cys. Consequently, this mutation resulted in a detectable increase in activity and thermostability, with minor activity (3.2%) after 30 min of incubation at 50 °C.

**Figure 3:**
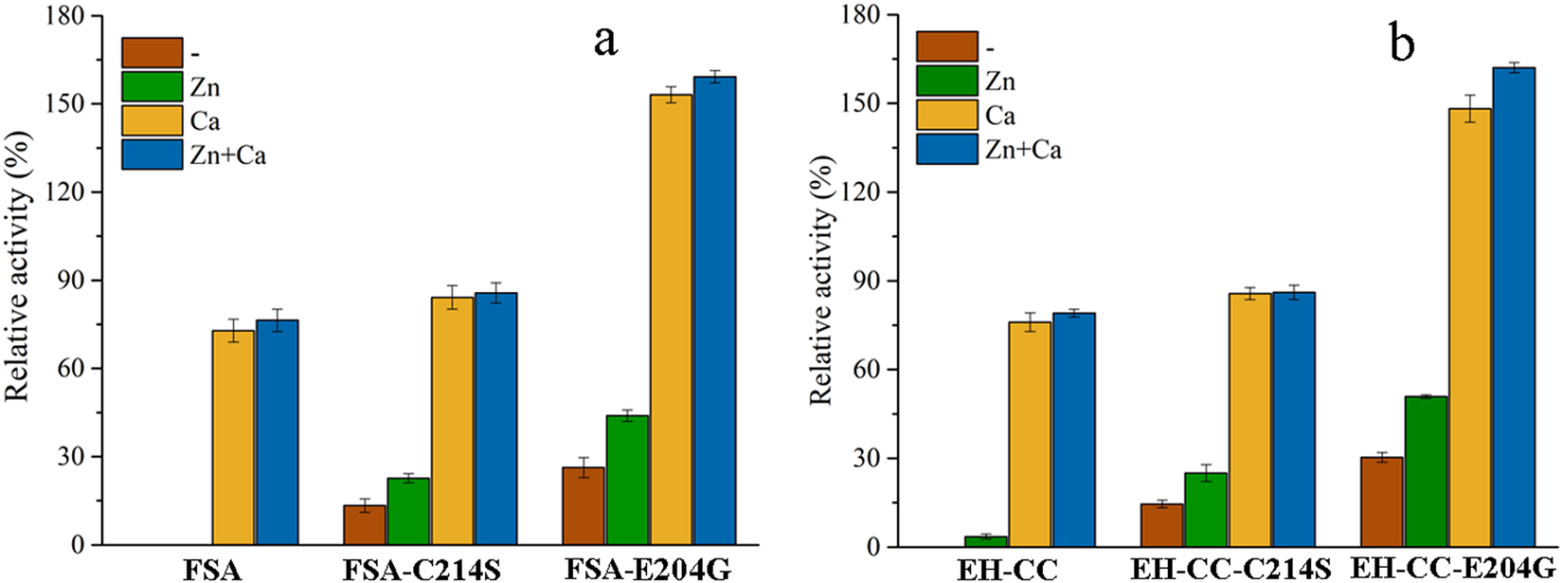
Relative activities of FSA and EH-CC variants in the presence of Ca^2+^ and Zn^2+^. (**a**) FSA and its mutants; (**b**) EH-CC and its mutants. The activity was measured after pre-incubating the starch-free reaction mixtures with 0.5 mM Ca^2+^, 0.01 mM Zn^2+^, or both ions, at 50 °C for 30 min. The activity of FSA in the absence of metal ions and without pre-incubation was set as 100%.

Further site-directed mutations involving this two-metal binding site were then evaluated, using wild-type FSA or mutant EH-CC as the template. When compared to the Zn^2+^-binding residues, site 214 displayed high conservation (Asp or Cys), but lacked subfamily specificity (Fig. 2). To alter Zn^2+^ binding in FSA, site Cys214 was replaced with Ser or Asp, and thereby four mutants were constructed from FSA and EH-CC. As a result, the replacement of Cys214 with the charged Asp led to the complete loss of enzyme activity (data not shown), despite a high level of protein expression (Figure S1). Conversely, high enzyme activity was retained after the replacement with Ser in FSA-C214S and EH-CC-C214S mutants, and thermostability was unexpectedly enhanced, with 13.4% and 9.3% residual activity detected after the 30-min incubation period. It is noteworthy that both C214S mutants exhibited obvious Zn^2+^-stimulated stability, and approximately 20% of the activity was retained by the addition of 0.01 mM Zn^2+^. In addition, the thermostability of these two C214S mutants was obviously enhanced by the presence of Ca^2+^, with higher residual activity (84.2% and 85.7%) in the FSA and EH-CC mutants (Fig. 3a and b).

**Figure 4:**
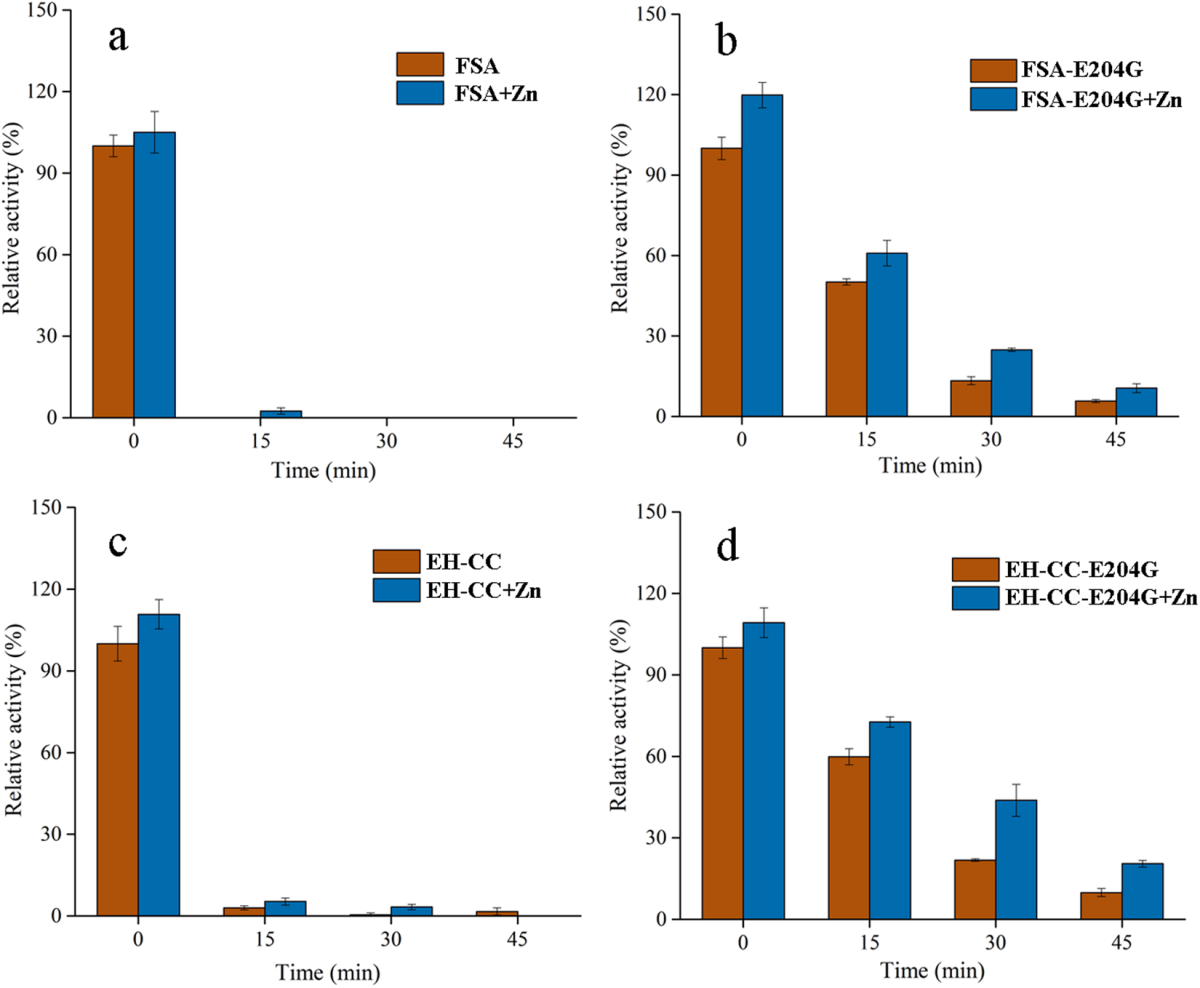
Influence of Zn^2+^ on the thermostability of FSA (**a**) and FSA-E204G (**b**), EH-CC (**c**) and EH-CC-E204G (**d**). The activity was measured after pre-incubating the starch-free reaction mixtures with 0.01 mM Zn^2+^ at 50 °C and sampled interval for activity measurement. The measured activity of FSA in the absence of metal ions and without pre-incubation was set as 100%.

In the alignment of the two-metal (Ca, Zn) center, all sequences of GH13_6 and GH13_7 α-amylases, except FSA, possessed a conserved Gly for Ca^2+^-binding (Fig. 2). The corresponding residue was replaced with Glu (G204) in the sequence of FSA. By site-directed mutagenesis, the replacement of Glu204 with Gly resulted in a remarkable increase in thermostability, with 50.2% and 59.9% activity retained in FSA-E204G and EH-CC-E204G, respectively, after 15 min of incubation in the absence of any metal ions (Fig. 4). Of note, the Ca^2+^-mediated stimulation of thermostability was greatly enhanced after the introduction of this mutation. Compared to its initial activity without the addition of any metal ion, FSA-E204G exhibited a remarkably high activity of 153.1% after 30 min of incubation at 50 °C in the presence of Ca^2+^. Furthermore, EH-CC-E204G exhibited a higher thermostability than that of FSA-E204G, implying that the mutant has a much more stable structure (Fig. 3b). Interestingly, Zn^2+^ had a positive effect on the constructs with a mutation at site 204, with 44.0% activity for FSA-E204G, and a higher residual activity (50.8%) in EH-CC-E204G after the 30-min incubation period.

### Differential scanning calorimetry (DSC) analysis of the thermal stability of FSA and EH-CC-E204G

**Figure 5:**
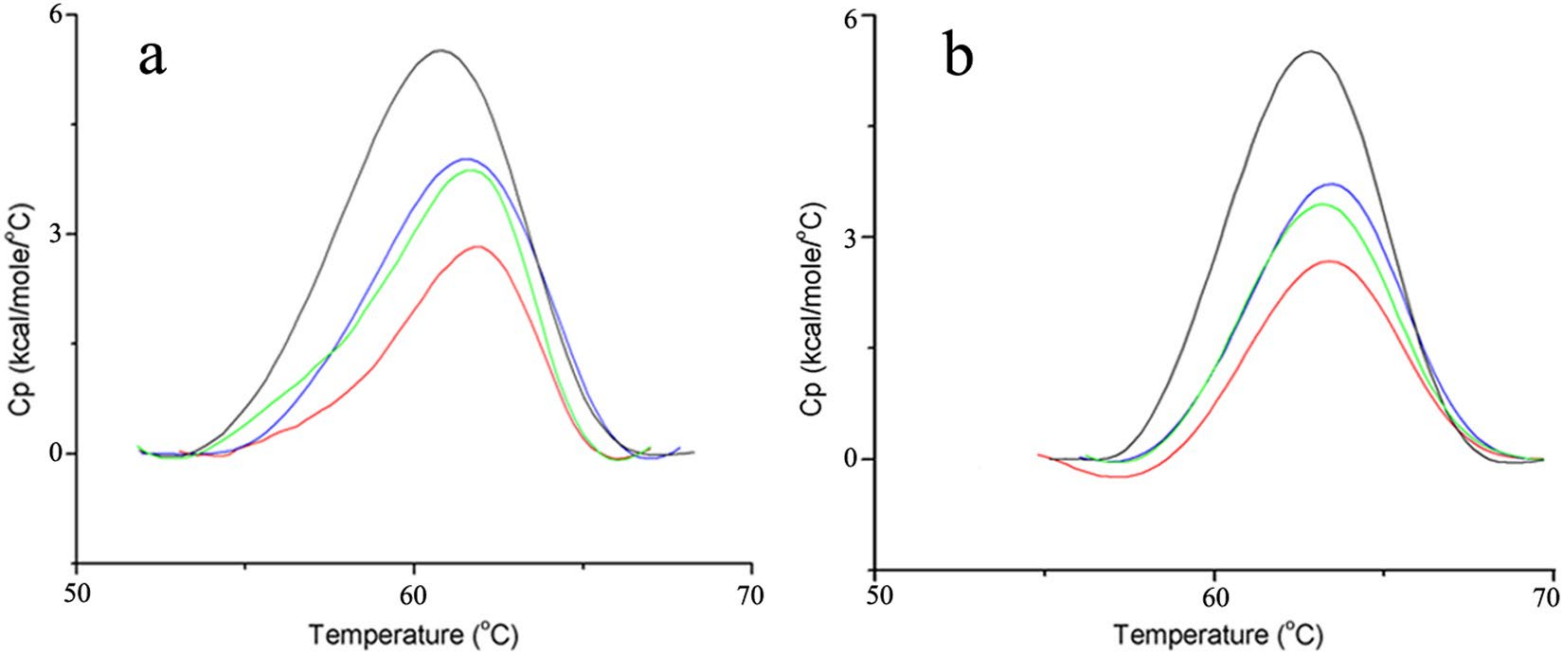
DSC spectrums of the purified FSA (**a**) and EH-CC-E204G (**b**). The scanning profile in the absence of metal ions (black line), in the presence of 0.5 mM Ca^2+^ (blue line), 0.01 mM Zn^2+^ (green line), and both ions (red line). Using the dialysis buffer as the reference, 0.8 mg ml^−1^ of FSA and EH-CC-204 were scanned from 20 to 90 °C.

DSC experiments were performed using purified FSA and EH-CC-E204G to quantitatively assess the effects of Ca^2+^ and Zn^2+^ on protein folding stability. DSC scans of each sample are shown in Fig. 5. Both proteins were stabilized by the addition of Ca^2+^ and Zn^2+^; an increase in the melting temperature (*T*_m_) from 60.5 °C to 61.3 °C was obtained for FSA in the presence of Ca^2+^, while the *T*_m_ value increased to 61.1 °C in the presence of Zn^2+^ (Table 1). In agreement with the enzyme thermostability data, FSA retained the highest thermostability in the presence of both ions (61.5 °C). Compared to wild-type FSA, the replacement of Glu with Gly in EH-CC resulted in an enhanced *T*_m_ value (62.7 °C), even without additional ions. Furthermore, supplementation with Ca^2+^ also enhanced the *T*_m_ value of EH-CC-E204G to 63.3 °C, whereas slightly weaker elevation was detected in the presence for Zn^2+^ (63.1 °C).

**Table 1:** *T*_m_ values of FSA and EH-CC-E204G determined by DSC analysis. “−” represents no metal ion addition, each sample were measured for three times and the systematic errors for these measurement are ± 0.2 °C.

	FSA (°C)	EH-CC-E204G (°C)
−	60.5	62.7
Zn^2+^	61.1	63.1
Ca^2+^	61.3	63.3
Ca^2+^ + Zn^2+^	61.5	63.4

### Isothermal titration calorimetry (ITC) analysis

**Figure 6:**
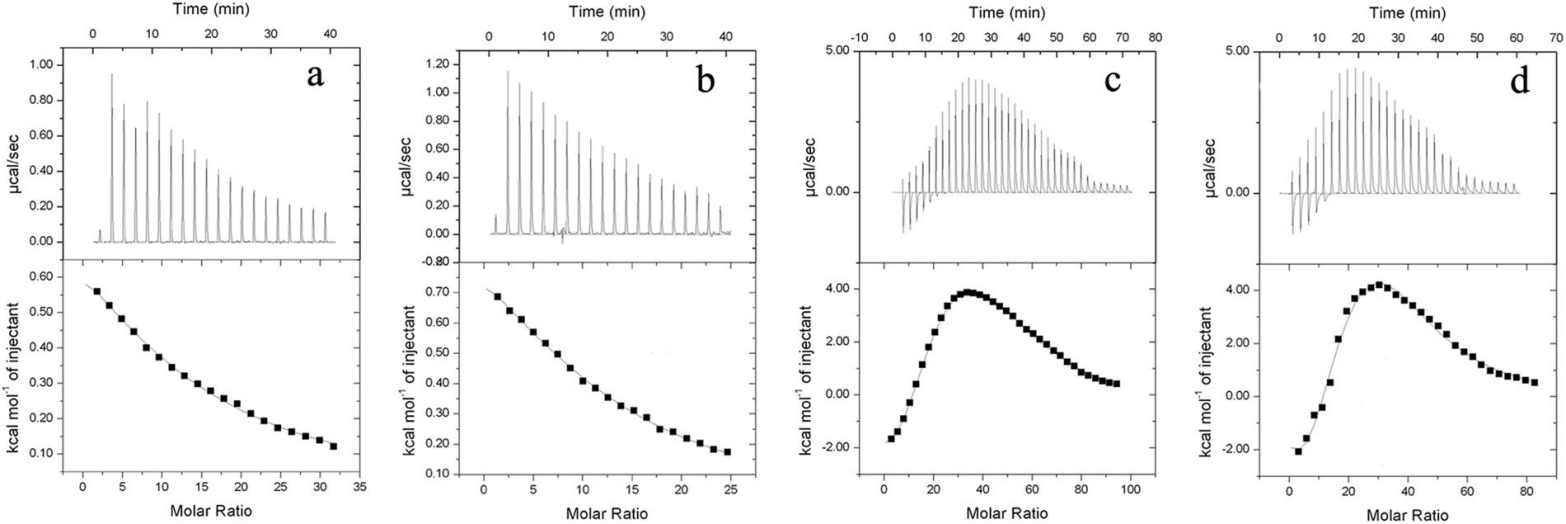
Isothermal titration microcalorimetric analysis of Ca^2+^ and Zn^2+^ binding to FSA (**a**,**c**) and EH-CC-E204G (**b**,**d**). Trace of the calorimetric titration of 20 × 2-μl aliquots of 15 mM CaCl_2_ or 30 (36) × 1-μl aliquots of 15 mM ZnCl_2_ into 50 μM proteins (top), and integrated binding isotherms (bottom).

Titration of 15 mM CaCl_2_ into FSA and EH-CC-E204G resulted in endothermic and monophasic isotherm. As shown in Fig. 6a and b, the best fit model for the ITC data was a one-set binding sites model showing that at least one Ca^2+^ binds enthalpically to FSA with a low affinity (*K*_d_ = 746.3 μM and Δ*H* = 1.3 kcal/mol), and a higher affinity was observed for EH-CC-E204G (*K*_d_ = 653.6 μM and Δ*H* = 1.2 kcal/mol).

The titration of ZnCl_2_ into these two proteins resulted in a multiphasic calorimetric isotherm that was best fit by the sequential binding model. In the isothermal comparison between FSA and EH-CC-E204G (Fig. 6c and d), it is noteworthy that one additional site (site 4) was observed in the titration profile of EH-CC-E204G, implying that this site might be the Zn^2+^-binding site in the two-metal region. This endothermic reaction showed a high dissociation constant for Zn^2+^ binding (*K*_*d4*_ = 1422.5 μM, Δ*H*_*4*_ = 260.8 kcal/mol), indicating that the created Zn^2+^-binding site can bind to Zn^2+^, but with low affinity. This is in agreement with the modest increased thermostability of those E204G mutants when in the presence of Zn^2+^. In addition to this novel binding site, the isotherm of Fig. 6c exhibited an initial exothermic phase (*K*_*d1*_ = 1642.0 μM, Δ*H*_*1*_ = −108.3 kcal/mol) representing stoichiometric Zn^2+^ binding to a low-affinity site, followed by a second endothermic, another low-affinity binding site. Subsequent Zn^2+^ binding was an endothermic reaction with modest affinity to site 3 (*K*_*d3*_ = 444.4 μM). The last phase (site 5) in FSA was an exothermic phase and exhibited a similar affinity to that of site 1 (Table 2).

**Table 2:** Thermodynamics of metal ions binding.

Metal ion	Site	Parameter	FSA	EH-CC-E204G
Ca^2+^	Site 1	*K*_*d*_ (μM)	746.3	653.6
		Δ*H* (kcal/mol)	1.3	1.2
Zn^2+^	Site 1	*K*_*d*_ (μM)	1642.0	724.6
		Δ*H* (kcal/mol)	−108.3	−64.2
	Site 2	*K*_*d*_ (μM)	2941.2	4524.9
		Δ*H* (kcal/mol)	185.4	6.9
	Site 3	*K*_*d*_ (μM)	444.4	96.2
		Δ*H* (kcal/mol)	242.1	67.7
	Site 4	*K*_*d*_ (μM)		1422.5
		Δ*H* (kcal/mol)		260.8
	Site 5	*K*_*d*_ (μM)	961.5	869.6
		Δ*H* (kcal/mol)	−159.4	−109.7

## Discussion

The identification of various features underlying the stability of thermostable enzymes remains a subject of ongoing study^18^. Mesophilic FSA from bacteria is evolutionary closely related to thermophilic archaeal PWA, but displays a sharply decreased thermostability, thus providing an ideal target for revealing strategies for the adaption of this mesophilic protein to a mesophilic environment. As an important signal for lateral gene transfer from PWA to FSA, the uncommon (Ca, Zn) two-metal center only exists in a few GH13_7 α-amylases, including PWA and those of some *Thermococcus* species^27,29,31,32^. By site-directed mutagenesis, both Ca^2+^- and Zn^2+^-binding sites were found to be important for the thermostability of PWA^27,28^. As these two sites are in close proximity, we suspected that they might function cooperatively in retaining the rigidity of the protein under higher temperature conditions.

To gain further insight into the function of this two-metal center, we first generated a mutation in EH-CC around the Zn^2+^-binding center by introducing a disulfide bond. This disulfide bond is adjacent to the Zn^2+^-binding site in PWA and rigidifies the active site area at high temperatures^27^. However, the molecular roles of this disulfide bond on thermostability of PWA remains to be experimental verified. In this study, a slightly enhanced thermostability observed in the EH-CC mutant implies that this disulfide bond contributes to the protein stabilization under restrict temperatures. Furthermore, the thermostability of EH-CC-derived proteins showed higher Zn^2+^-dependence than those of FSA and FSA-derived mutants. In the PWA structure, this disulfide bridge is in close vicinity to the zinc-binding site, these findings raise the possibility that this disulfide bond relating to the Zn^2+^ binding and ensuring a compact FSA structure. In PWA, besides the conserved Ca^2+^ located at the interface between domain A and B, another anomalous signal matching Zn^2+^ was detected near the Ca^2+^-binding site^27^. In contrast, the undetectable Zn^2+^ stimulation of FSA suggested that tight Zn^2+^-binding or no binding occurs in the FSA structure. Among three residues that act as Zn^2+^-binding residues, a mutation of Cys165 to Ser dramatically decreased the thermostability of PWA at 115 °C^28^. Conversely, the same mutation (C214S) in FSA not only resulted in an increased thermostability, but also enhanced the Zn^2+^-induced stimulation of thermostability. An explanation for this stimulation is that no Zn^2+^ binds to the wild-type FSA protein, and the replacement of Cys with Ser facilitates this binding. Meanwhile, the substitution of Cys214 with Asp completely abolished the activities of FSA and EH-CC, further demonstrating the important roles of this site in retaining the protein folding of FSA.

In the PWA structure, totally eight residues are binding amino acids involved in the two-metal center^27^. Based on a multiple alignment, we found only one residue (Gly204) was substituted by Glu in FSA. High conservation of this site in the α-amylases of GH13_6 and GH13_7 implies that this site is critical to the thermostability of these proteins. In this study, the replacement of Glu204 with Gly greatly enhanced the thermostability of EH-CC-E204G in the absence of any metal ions. In addition, the Ca^2+^-dependence of two E204G mutants became much higher than that of their parental proteins, demonstrating that mutations at this site may contribute to Ca^2+^-binding and further stabilize the protein. Interestingly, the modest enhanced Zn^2+^-stimulated thermostability, together with the appearance of a novel binding site after titration of Zn^2+^ into EH-CC-E204G, implied that the Zn^2+^ binding site was also resumed. This provides further evidence for the cooperative functions of this two-metal center in maintaining the protein rigidity and stability. However, both ion binding affinities revealed by ITC analysis are much lower, indicating that the pockets for Ca^2+^ and Zn^2+^ binding need to be further addressed. In FSA, we suspected that Ca^2+^ can bind to this two-metal region but with very low affinity, while no Zn^2+^ binding occurs at this site. Mutant EH-CC-E204G creates a relative compact binding pocket for Ca^2+^ and Zn^2+^ accommodation, and thereby gains an improved thermostability upon these two ions. However, in compared to PWA, these binding affinities are much lower and need to be improved in our future work.

In summary, most residues involving the (Ca, Zn) two-metal center in the thermophilic archaeal α-amylase PWA are also found in its mesophilic homolog, FSA. In the mesophilic environment, FSA retained most of its residues for ion binding but lost its thermostability during evolution. Mutations leading to the reconstruction of this two-metal center suggested that these two ion binding sites have synergistic effects. As most α-amylases contain a conserved Ca^2+^, this synergic cooperation provides a basis for designing engineered α-amylases with desired thermal properties, by *in silico* rational design and the systems engineering of a Zn^2+^-binding site adjacent to the Ca^2+^-binding site.

## Methods

### Bacterial strains, plasmids, and materials

*Escherichia coli* DH5α was used for plasmid construction and *E. coli* BL21-CodonPlus was used to express FSA and mutants. Unless otherwise indicated, recombinant strains were cultured in the Luria-Bertani (LB) medium consisting of 1% tryptone, 0.5% yeast extract, and 1% NaCl (pH 7.0).

### Sequence alignment and mutant construction

Some α-amylases encoding sequences from GH13_5, 13_6, and 13_7 subfamilies were extracted from NCBI and aligned by ClustalX^33^. Gene splicing by overlap extension PCR (SOE-PCR) was used for site-directed mutagenesis^34^. All the primers used for mutant generating were listed in Table S1. According to the corresponding sequence of PWA, Glu200 and His201 in FSA were replaced by Cys, which resulted in a double mutant designated as EH-CC. Then, other mutants involving in Ca^2+^ or Zn^2+^ binding site were generated on the basis of FSA or EH-CC, respectively. The PCR product was ligated into pGM-18T vector (Promega), and transformed into *E. coli* 5α for sequencing. Correct plasmid was digested, ligated into the expression vector pETDuet-1, and transformed into *E. coli* BL21-CodonPlus for protein expression.

### Protein expression and purification

The *E. coli* BL21-CodonPlus recombinants were cultivated in LB medium with 100 μg ml^−1^ ampicillin and 40 μg ml^−1^ chloromycetin. When reaching at the mid-exponential growth phase, cells were induced by isopropyl-β-Dthiogalactopyranoside (IPTG) at a final concentration of 1 mM. After incubated at 16 °C, cells were harvested, washed and resuspended as we previously described^14^. For enzyme activity assay, the purified proteins were prepared by passing through a His-trap column. For DSC analysis, proteins with high purity were obtained by a three-step purification procedure^14^. The resulting protein fractions were analyzed by 12% SDS-PAGE. The protein concentration was determined by coomassie brilliant method.

### Influences of Ca^2+^ and Zn^2+^ on the α-amylase’s activity and thermal stability

α-Amylase activity was determined by measuring the amount of reducing sugar released during the enzymatic hydrolysis of 5 g l^−1^ of soluble starch in 50 mM PBS (pH 6.0) at 50 °C for 15 min. Reducing sugar was measured by a modified dinitrosalicylic acid method^35^. One unit of α-amylase activity was defined as the amount of enzyme that released 1 μmol of reducing sugar as glucose per minute under the assay conditions^36^. For thermostability assay, enzymes were diluted to a final concentration of 10 μg mg^−1^ in buffer A and the assay mixtures were incubated at 50 °C for 30 min. The residual activity was measured as standard procedures described above, in the presence or absence of indicated concentrations of Ca^2+^ and (or) Zn^2+^.

### DSC analysis for thermal stability

Proteins from Superdex-200 were dialyzed overnight in buffer consisting of 20 mM Hepes, and concentrated by ultrafiltration. After degassing by stirring under vacuum prior to scanning, samples in the MicroCal VP-DSC (Malven) were cooled down to 20 °C and gradually heated, at a scan rate of 1.5 °C min^−1^. Using the dialysis buffer as a baseline, 0.8 mg ml^−1^ of FSA and EH-CC-204 were scanned from 20 to 90 °C, in the absence or presence of 0.5 mM Ca^2+^, 0.01 mM Zn^2+^, or both ions. Using the Origin software from MicroCal Inc, the thermal midpoints (*T*_m_) was analyzed by subtracting the baseline.

### ITC analysis for Ca^2+^ and Zn^2+^ binding

ITC experiments were performed with a MicroCal VP-ITC microcalorimeter. The sample of FSA or EH-CC-E204G was placed in the sample cell, and the corresponding dialysis buffer was placed in the reference cell. The titrations CaCl_2_ and ZnCl_2_ solutions were prepared with the dialysis buffer and degassed in a ThermoVac apparatus (Microcal). Titrations were performed at 25 °C with protein concentrations between 50 and 60 μM. The titration isotherm was integrated by using the Origin software provided by Microcal Inc.

### Data availability

All data generated or analyzed during this study are included in this published article (and its Supplementary Information files).

## Electronic supplementary material

Supplementary information

## References

[1] Chakravarty S, Varadarajan R (2002). Elucidation of factors responsible for enhanced thermal stability of proteins: a structural genomics based Study. Biochemistry.

[2] England JL, Shakhnovich BE, Shakhnovich EI (2003). Natural selection of more designable folds: a mechanism for thermophilic adaptation. Proc. Natl. Acad. Sci..

[3] Sammond DW (2016). Comparing residue clusters from thermophilic and mesophilic enzymes reveals adaptive mechanisms. PLoS ONE.

[4] Chen J, Stites WE (2004). Replacement of staphylococcal nuclease hydrophobic core residues with those from thermophilic homologs indicates packing is improved in some thermostable proteins. J. Mol. Biol..

[5] Gromiha MM, Pathak MC, Saraboji K, Ortlund EA, Gaucher EA (2013). Hydrophobic environment is a key factor for the stability of thermophilic proteins. Proteins.

[6] Vogt G, Woell S, Argos P (1997). Protein thermal stability, hydrogen bonds, and ion pairs. J. Mol. Biol..

[7] Berezovsky IN, Shakhnovich EI (2005). Physics and evolution of thermophilic adaptation. Proc. Natl. Acad. Sci..

[8] Radestock S, Gohlke H (2011). Protein rigidity and thermophilic adaptation. Proteins: Struct. Funct. Bioinf..

[9] de Souza PM, Magalhues PO (2010). Application of microbial α-amylase in industry–a review. Brazilian J. Microbiol..

[10] Cantarel BL (2009). The Carbohydrate-Active EnZymes database (CAZy): an expert resource for glycogenomics. Nucleic. Acids Res..

[11] Majzlová K, Pukajová Z, Janeček S (2013). Tracing the evolution of the α-amylase subfamily GH13_6 covering the amylolytic enzymes intermediate between oligo-1,6-glucosidases and neopullulanases. Carbohydr. Res..

[12] Stam MR, Danchin EG, Corinne R, Coutinho PM, Henrissat B (2006). Dividing the large glycoside hydrolase family 13 into subfamilies: towards improved functional annotations of α-amylase-related proteins. Protein Eng. Des. Sel..

[13] Janeček S, Svensson B, MacGregor EA (2014). α-Amylase: an enzyme specificity found in various families of glycoside hydrolases. Cell. Mol. Life Sci..

[14] Li CF (2014). Close relationship of a novel *Flavobacteriaceae* α-amylase with archaeal α-amylases and good potentials for industrial applications. Biotechnol. Biofuels.

[15] Janeček S (2009). Amylolytic enzymes-focus on the alpha-amylases from archaea and plants. Nova Biotechnol..

[16] Janeček S (1994). Sequence similarities and evolutionary relationships of microbial, plant and animal α-amylases. Eur. J. Biochem..

[17] Godány A, Majzlová K, Horváthová V, Janeček S (2010). Tyrosine 39 of GH13 α-amylase from *Thermococcus hydrothermalis* contributes to its thermostability. Biologia.

[18] Prakash O, Jaiswal N (2010). α-Amylase: an ideal representative of thermostable enzymes. Appl. Biochem. Biotechnol..

[19] Alikhajeh J (2010). Structure of *Bacillus amyloliquefaciens* α-amylase at high resolution: implications for thermal stability. Acta Crystallogr. Sect. F: Struct. Biol. Cryst. Commun..

[20] Machius M, Wiegand G, Huber R (1995). Crystal Structure of Calcium-depleted *Bacillus licheniformis* α-amylase at 2.2 Å Resolution. J. Mol. Biol..

[21] Kagawa M, Fujimoto Z, Momma M, Takase K, Mizuno H (2003). Crystal structure of *Bacillus subtilis* alpha-amylase in complex with acarbose. J. Bacteriol..

[22] Janecĕk S (1997). alpha-Amylase family: molecular biology and evolution. Prog. Biophys. Mol. Biol..

[23] Boel E (1990). Calcium binding in alpha-amylases: an X-ray diffraction study at 2.1-Å resolution of two enzymes from *Aspergillus*. Biochemistry.

[24] Koch R, Zablowski P, Spreinat A, Antranikian G (1990). Extremely thermostable amylolytic enzyme from the archaebacterium *Pyrococcus furiosus*. FEMS Microbiol. Lett..

[25] Nonaka T (2003). Crystal structure of calcium-free α-amylase from *Bacillus* sp. strain KSM-K38 (AmyK38) and its sodium ion binding sites. J. Biol. Chem..

[26] Sajedi RH, Taghdir M, Naderimanesh H, Khajeh K, Ranjbar B (2007). Nucleotide sequence, structural investigation and homology modeling studies of a Ca^2+^-independent alpha-amylase with acidic pH-profile. J. Biochem. Mol. Biol..

[27] Linden A, Mayans O, Meyer-Klaucke W, Antranikian G, Wilmanns M (2003). Differential regulation of a hyperthermophilic α-amylase with a novel (Ca, Zn) two-metal center by zinc. J. Biol. Chem..

[28] Savchenko A, Vieille C, Kang S, Zeikus JG (2002). *Pyrococcus furiosus* α-amylase is stabilized by calcium and zinc. Biochemistry.

[29] Cheng HX, Luo ZD, Lu MS, Wang SJ (2017). The hyperthermophilic α-amylase from *Thermococcus*, sp. HJ21 does not require exogenous calcium for thermostability because of high-binding affinity to calcium. J. Microbiol..

[30] Janeček S, Lévêque E, Belarbi A, Haye B (1999). Close evolutionary relatedness of α-amylases from archaea and plants. J. Mol. Evol..

[31] Leveque E, Haye B, Belarbi A (2000). Cloning and expression of an alpha-amylase encoding gene from the hyperthermophilic archaebacterium *Thermococcus hydrothermalis* and biochemical characterization of the recombinant enzyme. FEMS Microbiol. Lett..

[32] Lim JK (2007). Critical factors to high thermostability of an α-amylase from hyperthermophilic archaeon *Thermococcus onnurineus* NA1. J. Microbiol. Biotechnol..

[33] Thompson JD, Gibson TJ, Plewniak F, Jeanmougin F, Higgins DG (1997). The CLUSTAL_X windows interface: flexible strategies for multiple sequence alignment aided by quality analysis tools. Nucleic. Acids. Res..

[34] Horton RM, Hunt HD, Ho SN, Pullen JK, Pease LR (1989). Engineering hybrid genes without the use of restriction enzymes: gene splicing by overlap extension. Gene.

[35] Miller GL (1959). Use of dinitrosalicylic acid reagent for determination of reducing sugar. Anal. Chem..

[36] Hagihara H (2001). Novel α-amylase that is highly resistant to chelating reagents and chemical oxidants from the alkaliphilic *Bacillus* isolate KSM-K38. Appl. Environ. Microbiol..

