# Supplementary material for: Functional and cooperative stabilization of a two-metal (Ca, Zn) center in α-amylase derived from Flavobacteriaceae species
Source: Sci Rep. 2017 Dec 20;7:17933. doi: 10.1038/s41598-017-18085-4 (PMC5738361; doi:10.1038/s41598-017-18085-4)
Supplement: Supplementary file 1 — Supplementary information [file 41598_2017_18085_MOESM1_ESM.pdf]

Functional and cooperative stabilization of a two-metal (Ca, Zn) center in α-amylase derived from *Flavobacteriaceae* species

Huijia Yin, Zhou Yang, Xinyu Nie, Shannan Li, Xuyang Sun, Chao Gao, Zenghang Wang, Guangming Zhou, Ping Xu, Chunyu Yang*

State Key Laboratory of Microbial Biotechnology, Shandong University, Jinan 250100, People's Republic of China

* Correspondence author: Chunyu Yang

Email: ycy21th@sdu.edu.cn

Table S1 Primers for FSA and its mutants. Restriction sites are indicated in underline.

Primer	Sequence (5` to 3` direction)
FSA F	5’-CGGGATCCGGATGACAATAATAATTATAC-3’
FSA R	5’- GACGTCGACTTACTCTCCCGAAACC-3’
EH-CC F	5’-AGTTTGCATTGTTGTGATGAGGAAGCACTT-3’
EH-CC R	5’-TTCCTCATCACAACAATGCAAACTGTTGG-3’
EH-CC-E204G F	5’-GCATTGTTGTGATGAGGGAGCACTTTTCTTCG-3’
EH-CC-E204G R	5’-GTGCTCCCTCATCACAACAATGCAAACTG-3’
FSA-E204G F	5’-GCATGAGCATGATGAGGGAGCACTTTTCTTCG-3’
FSA-E204G R	5’-GTGCTCCCTCATCATGCTCATGCAAACTG-3’
C214D F	5’-GACCTTGACCATCATCAGCCCCG-3’
C214D R	5’-GCTGATGATGGTCAAGGTCCTGTTC-3’
C214S F	5’-GAACAGGACCTTTCCCATCATCAGCCCCG-3’
C214S R	5’-GCTGATGATGGGAAAGGTCCTGTTCCTCGA-3’

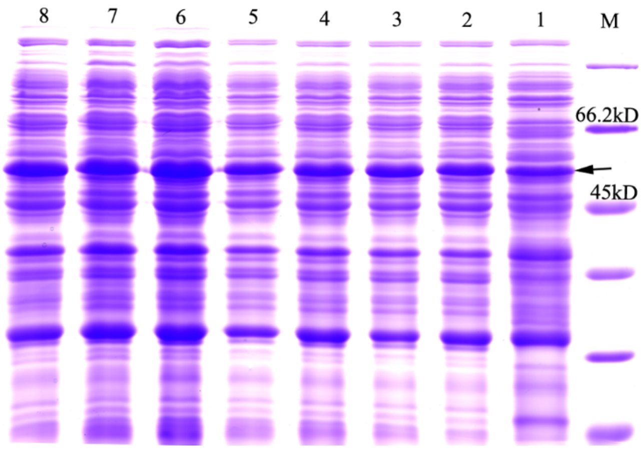

Figure S1. SDS-PAGE spectrum of FSA and mutated proteins. **M**, marker; **1**, FSA; **2**, EH-CC; **3**, FSA-E204G; **4**, EH-CC-E204G; **5**, FSA-C214D; **6**, FSA-C214D; **7**, FSA-C214S; **8**, EH-CC-C214S.
